# CDSKNN^XMBD^: a novel clustering framework for large-scale single-cell data based on a stable graph structure

**DOI:** 10.1186/s12967-024-05009-w

**Published:** 2024-03-03

**Authors:** Jun Ren, Xuejing Lyu, Jintao Guo, Xiaodong Shi, Ying Zhou, Qiyuan Li

**Affiliations:** 1 School of Informatics, Xiamen University, Xiamen, 361105 China; 2 Department of Hematology, The First Affiliated Hospital of Xiamen University and Institute of Hematology, School of Medicine, Xiamen University, Xiamen, 361102 China; 3 National Institute for Data Science in Health and Medicine, School of Medicine, Xiamen University, Xiamen, 361102 China

**Keywords:** scRNA-seq, Clustering, Large-scale, Imbalance ratio

## Abstract

**Background:**

Accurate and efficient cell grouping is essential for analyzing single-cell transcriptome sequencing (scRNA-seq) data. However, the existing clustering techniques often struggle to provide timely and accurate cell type groupings when dealing with datasets with large-scale or imbalanced cell types. Therefore, there is a need for improved methods that can handle the increasing size of scRNA-seq datasets while maintaining high accuracy and efficiency.

**Methods:**

We propose CDSKNN^XMBD^ (Community Detection based on a Stable K-Nearest Neighbor Graph Structure), a novel single-cell clustering framework integrating partition clustering algorithm and community detection algorithm, which achieves accurate and fast cell type grouping by finding a stable graph structure.

**Results:**

We evaluated the effectiveness of our approach by analyzing 15 tissues from the human fetal atlas. Compared to existing methods, CDSKNN effectively counteracts the high imbalance in single-cell data, enabling effective clustering. Furthermore, we conducted comparisons across multiple single-cell datasets from different studies and sequencing techniques. CDSKNN is of high applicability and robustness, and capable of balancing the complexities of across diverse types of data. Most importantly, CDSKNN exhibits higher operational efficiency on datasets at the million-cell scale, requiring an average of only 6.33 min for clustering 1.46 million single cells, saving 33.3% to 99% of running time compared to those of existing methods.

**Conclusions:**

The CDSKNN is a flexible, resilient, and promising clustering tool that is particularly suitable for clustering imbalanced data and demonstrates high efficiency on large-scale scRNA-seq datasets.

**Supplementary Information:**

The online version contains supplementary material available at 10.1186/s12967-024-05009-w.

## Background

The advancement of single-cell RNA sequencing (scRNA-seq) technology has propelled the development of single-cell data analysis methods, which is one of the key steps during the unsupervised clustering of cells based on gene expression patterns [1–3]. The quality of the clustering outcomes profoundly influences the credibility of subsequent analyses, including but not limited to cell type annotation, cell lineage inference, and the construction of cell developmental trajectories, playing a critical role in revealing the heterogeneity and diversity among cells [4, 5].

Numerous mature clustering methods for single-cell data have emerged from prior research efforts. In addition to widely used community detection algorithms such as louvain [6] and leiden [7], there are also SC3 [8], SIMLR [9], CIDR [10], and SAFE clustering [11], each of which addresses the construction of clustering frameworks from various perspectives, including iterative optimization, different information representations, missing value imputation, and similarity matrices between cells [12]. Nevertheless, the surge in sequencing depth and the expansion of throughput coverage have led to a proportional increase in the size of gene expression matrices [13]. Implementing the aforementioned clustering methods for million-cell datasets often encounters challenges, including time-intensive procedures, exceedingly high computational complexity, and the necessity for a well-configured computing environment. Furthermore, manually selected clustering outcomes frequently demonstrate pronounced subjectivity, compelling most methodologies [14, 15] to prioritize the adoption of clustering quality evaluation metrics such as the Calinski–Harabasz (CH) [16] and Gap-Statistic [17] indices to determine the optimal clustering results. However, challenges persist in managing datasets of this magnitude, particularly concerning issues related to protracted computational time.

Consequently, clustering frameworks have been developed specifically for large single-cell datasets. Notably, phenograph [18] employs the Jaccard similarity coefficient [19] to construct a similarity matrix for the K-Nearest Neighbor (KNN) graph [20] structure, subsequently employing the louvain algorithm for clustering; PARC [21] uses accelerated fine community partitioning to analyze phenotypes without resampling; FlowGrid [22] integrates DBSCAN [23] with grid-based methods, enhancing the robustness and scalability of DBSCAN for clustering extensive datasets, and selects the optimal parameter configuration using the CH index. Undeniably, the incorporation of graph-based clustering algorithms has emerged as the prevailing trend for clustering large-scale single-cell data. However, these methods often exhibit inconsistent performance, with room for improvement in terms of clustering scalability and robustness in diverse application scenarios [3, 24]. FlowGrid's clustering framework exhibits unstable performance concerning varying feature quantities of the data; phenograph encounter difficulties when dealing with highly imbalanced data, and although PARC exhibits high efficiency, uncertainties persist regarding clustering precision [24, 25].

To address these difficulties, we propose the CDSKNN^XMBD^, a novel single-cell clustering framework (CDSKNN: Community Detection based on a Stable K-Nearest Neighbor Graph) (XMBD: Xiamen Big Data, a biomedical open software initiative in the National Institute for Data Science in Health and Medicine, Xiamen University, China.). It combines partition clustering algorithm and community detection algorithm with the following steps: (i) conduct preliminary data partitioning using the mbkmeans [26] algorithm, along with outlier detection and removal in each partitioned region; (ii) randomly sample in each partitioned region and construct KNN graph structures under different $K$ values, employing community detection algorithms and applying Normalized Reduce Mutual Information [27] across multiple samplings to identify a stable graph structure; and (iii) perform louvain clustering based on the optimal graph structure, determine the optimal clustering resolution using the CH index, and map it to the entire dataset.

On the basis of the CDSKNN, we conducted a detailed evaluation of its clustering performance and compared it with that of the current mainstream clustering frameworks for large-scale single-cell data, including PARC, FlowGrid, and phenograph. In highly imbalanced cellular population scenarios, CDSKNN demonstrates outstanding clustering accuracy. It consistently provides precise cell type estimation, fully aligning with the gold standard and outperforming other clustering frameworks. Additionally, across datasets with diverse biological backgrounds, CDSKNN exhibits exceptional adaptability and demonstrates superior computational efficiency when handling large-scale datasets. Finally, compared to existing clustering frameworks, CDSKNN exhibits more stable clustering performance across different feature quantities, effectively balancing computational efficiency and clustering precision.

## Methods

### Algorithm design

#### Overview of the CDSKNN workflow

**Fig. 1:**
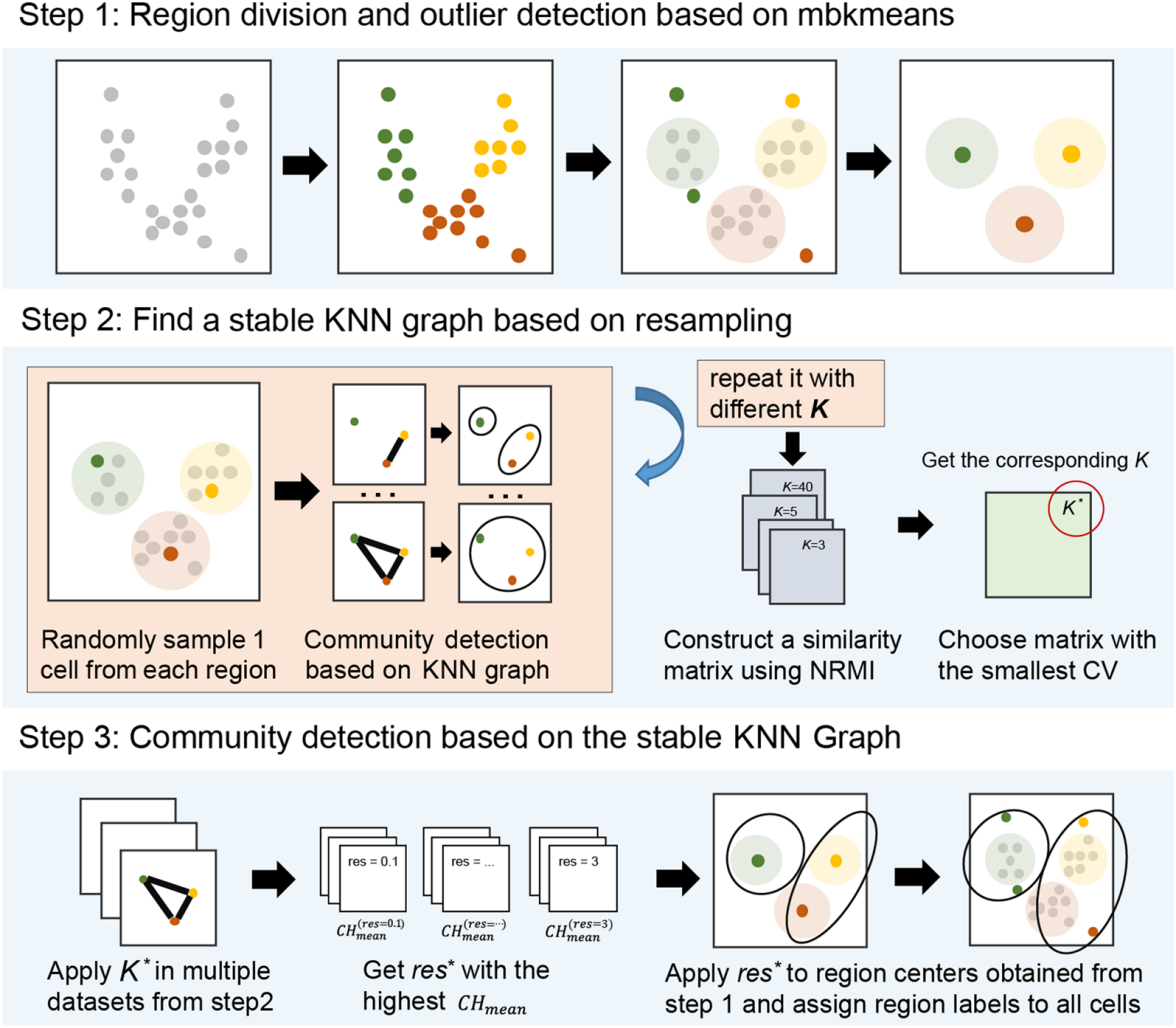
Overview of the CDSKNN workflow. The enabling features include region division and outlier detection, finding a stable KNN graph, and community detection according to the CH index

CDSKNN leverages three interconnected modules for the effective clustering of single-cell data (Fig. 1). First, it involves an initial partitioning of the data along with the identification of outliers within distinct regions. Second, resampling techniques are employed across various regions to construct a KNN graph structure to assess the stability of the network and determine the optimal structure $K$. Third, the construction of a stable KNN graph involves utilizing centroids from the initially demarcated regions, followed by the application of the louvain clustering algorithm across various resolutions, while the optimal resolution is determined by the Calinski-Harabasz (CH) index. Finally, the final clustering outcomes are projected back to all cells.

#### Region division and outlier detection based on mbkmeans

A. We partition the data into coarse-grained regions using mbkmeans [26], which is an improvement of K-means that processes small batches of data subsets in batches to reduce the computation time. Assuming we have $P$ cells, the data are preprocessed to yield a $P\times N$ matrix ${\varvec{M}}$, where $N$ represents the number of features. By employing mbkmeans, ${\varvec{M}}$ is partitioned into $g$ regions. For any given region $g$, it contains cells ${{\varvec{m}}}_{l}^{\left(g\right)}\in \{{{\varvec{m}}}_{1}^{\left(g\right)},{{\varvec{m}}}_{2}^{\left(g\right)},\dots ,{{\varvec{m}}}_{n}^{\left(g\right)}\}$, with the centroid denoted as ${{\varvec{C}}}^{(g)}$.

b. Based on the region partition, we employ the Mahalanobis distance [28] to examine the outliers of an arbitrary region $g$. Unlike the Euclidean distance, the Mahalanobis distance is independent of the measuring units and takes into account the correlation between dimensions. The distance between any point ${{\varvec{m}}}_{l}^{\left(g\right)}$ and the regional center ${{\varvec{C}}}^{(g)}$ is as follows:

1 $$D\left({{\varvec{m}}}_{l}^{\left(g\right)},{{\varvec{C}}}^{\left(g\right)}\right)=\sqrt{{\left({{\varvec{m}}}_{l}^{\left(g\right)}-{{\varvec{C}}}^{\left(g\right)}\right)}^{T}{\Sigma }^{-1}\left({{\varvec{m}}}_{l}^{\left(g\right)}-{{\varvec{C}}}^{\left(g\right)}\right)}$$

For high-dimensional datasets, the squared Mahalanobis distance follows a chi-squared distribution with degrees of freedom *P*.

2 $${D}^{2}\left({{\varvec{m}}}_{l}^{\left(g\right)},{{\varvec{C}}}^{\left(g\right)}\right)\sim {\chi }^{2}\left(P\right)$$

Through a hypothesis test, for any given significance level $\alpha$, the corresponding quantile is ${t}_{\alpha }$; then,

3 $$Pr\left({D}^{2}\left({{\varvec{m}}}_{l}^{\left(g\right)},{{\varvec{C}}}^{\left(g\right)}\right)<{t}_{\alpha }\right)=1-\alpha$$

If the Mahalanobis distance of any point ${{\varvec{m}}}_{l}^{\left(g\right)}$ to the center point ${{\varvec{C}}}^{\left(g\right)}$ cannot fulfill the Eq. (3), suggesting a significant difference, then it can be considered an outlier.

c. The regional center point is updated to ${({{\varvec{C}}}^{\boldsymbol{*}})}^{(g)}$ after outlier elimination of each region.

#### Finding a stable KNN graph based on resampling

The louvain algorithm is widely used in single-cell analysis and is capable of partitioning large-scale graphs into communities without specifying the number of communities. Its performance heavily relies on the underlying graph structure, which is commonly constructed using the KNN approach. Here, $K$ represents the number of closest neighbors used to represent each data point. An ideal $K$ can be obtained by all kinds of heuristic approaches. We use the following steps:

- Sample a point ${{\varvec{m}}}_{l}^{(g)}$ in each region, thereby creating a new matrix ${{\varvec{M}}}^{*}=\{{{\varvec{m}}}_{l}^{\left(1\right)},{{\varvec{m}}}_{l}^{\left(2\right)},\dots ,{{\varvec{m}}}_{l}^{(g)}\}$.
- K-nearest neighbor (KNN) graph structures were built with different $K$ ($K=\{{k}_{1},{k}_{2},\dots ,{k}_{z}\}$) values on the basis of ${{\varvec{M}}}^{\boldsymbol{*}}$. Run louvain community detection with default resolution ($res$). We obtain ${\text{z}}$ clustering results ${\varvec{U}}=\{{{\varvec{u}}}_{1},{{\varvec{u}}}_{2},\cdots ,{{\varvec{u}}}_{z}\}$.
- Repeat a-b $L$ times. We suppose that if a graph structure built on a $K$ value is stable enough, then the similarity of the clustering results produced by that $K$ value in $L$ resampling ought to be very high. Therefore, we use the normalized reduce mutual information (NRMI) [27] to construct the similarity matrix ${{\varvec{s}}}_{{\varvec{k}}}$ of the clustering results based on the same $K$ value. Reduce mutual information (RMI) is improved on the basis of mutual information (MI) [29]. In some cases, the problem of assigning high mutual information values to completely different partitions can be resolved by the RMI. For example, if the clustering result is made up of a single vertex cluster, MI will consider the result to be very stable, which is undesirable. The following is a definition of the RMI.
  4 $$RMI(\varvec{u}_{i};\varvec{u}_{j}) = I\left( {{\varvec{u}}_{i} ;{\varvec{u}}_{j} } \right) - \frac{1}{P}\log \Omega \left( {a,b} \right)$$

where $\Omega (a,b)$ is an integer equal to the number of $P\times N$ nonnegative integer matrices with row sums $a=\{{a}_{{u}_{i}}\}$ and column sums $b=\{{b}_{{u}_{j}}\}$. Furthermore, the normalized RMI is defined as follows:

5 $$NRMI({\varvec{u}}_{i} ;{\varvec{u}}_{j}) = \frac{{RMI({\varvec{u}}_{i} ;{\varvec{u}}_{j}) }}{{\frac{1}{2} \times \left[ {RMI({\varvec{u}}_{i} ;{\varvec{u}}_{j}) + RMI({\varvec{u}}_{i} ;{\varvec{u}}_{j} )} \right]}}$$

d. Calculate the coefficient of variation (CV) [30] of any similarity matrix ${{\varvec{s}}}_{{\varvec{k}}}$, choose the matrix with the minimal CV, and the corresponding $K$ is a stable $K$, i.e., ${K}^{*}$.

#### Community detection based on a stable ${\mathbf{K}}^{\mathbf{*}}\mathbf{N}\mathbf{N}$ graph

- Construct the ${K}^{*}NN$ graph structure through ${({{\varvec{M}}}^{*})}_{l}$ produced by $L$ resampling of ${K}^{*}$; then, run the louvian clustering algorithm under $t$ different resolutions;
- For the $L$ clustering results obtained at a specific resolution; we utilize the CH index to assess the stability of the clusters. The result with the highest CH means value among all the resolutions, denoted as ${res}^{*}$, is chosen as the optimal result.
- Finally, ${res}^{*}$ is mapped back to all cells according to the region dividing label to obtain the final clustering label.

### Validation datasets

#### scRNA-seq data

To highlight the advantages of CDSKNN, we collected two groups of scRNA-seq datasets from previous literature (Table 1). The first group of data originates from single-cell atlases of human fetal tissues [31] and encompasses 15 organs, with cell numbers ranging from 8,000 to 1.7 million across different organs. We conducted robustness testing of the CDSKNN parameters using these datasets and evaluated the adaptability of the different methods to imbalanced data. The second group of datasets included multiple single-cell datasets from various studies [13, 31–38]; employed diverse library preparation methods; and involved different tissues from humans or mice, such as the hypothalamus, peripheral blood, and heart, with cell numbers ranging from 8,000 to 1.46 million. We assessed the universality and operational efficiency of CDSKNN using these datasets.

**Table 1:** General information on the validation single-cell sequencing datasets

Group	Accession	Species	Tissue	Cell Number	Cell Type Number	Refs.
**Group 1** **GSE156793**	Thymus	Human	thymus	8,779	5	[31]
	Stomach		stomach	12,106	16	
	Spleen		spleen	13,180	9	
	Placenta		placenta	29,876	12	
	Muscle		muscle	30,872	11	
	Pancreas		pancreas	45,653	14	
	Intestine		intestine	51,650	12	
	Eye		eye	51,836	16	
	Heart		heart	101,749	16	
	Liver		liver	113,138	9	
	Kidney		kidney	155,386	9	
	Lung		lung	217,738	13	
	Adrenal		adrenal	387,771	12	
	Cerebellum		cerebellum	1,092,000	9	
	Cerebrum		cerebrum	1,751,246	9	
**Group 2**	GSE111107	Mouse	kidney glomeruli	12,954	5	[32]
	GSE102827	Mouse	visual cortex	48,266	8	[35]
	GSE131907	Human	lung	180,069	7	[36]
	SCP1162	Human	colorectal	370,115	7	[38]
	SCP795	Human	lobules	611,034	18	[39]
	PRJEB38269	Human	iPSC, neurons	1,027,398	12	[34]
	MERFISHData	Mouse	neurons	1,027,848	16	[33]
	GSE158055	Human	PBMC*, BALF*	1,462,702	12	[13]

^*^PBMC: Peripheral Blood Mononuclear Cells; BALF: Bronchoalveolar Lavage Fluid

All the datasets contained labels pertaining to the cell types, facilitating the comparison of clustering performance. The majority of the data were downloaded from the Gene Expression Omnibus (GEO) website (https://www.ncbi.nlm.nih.gov/geo/) and the Single Cell Portal (SCP) website (https://singlecell.broadinstitute.org/single_cell).

#### Data preprocessing

We preprocessed the various data before validating the method with the data. As the single-cell datasets grow larger, additional processing factors, such as batch effects and identification of highly variable genes, need to be taken into account. To reduce the impact of validation by other factors, we preprocessed each dataset using the original study's processing steps, which included gene filtering, normalization, and screening for highly variable genes. Like most methods, we choose to perform clustering based on principal component analysis (PCA) [40]. As a one-click clustering framework, the default number of principal components (PCs) used is 5 if not specified by the user. It is worth noting that some of the datasets had published preprocessing results, which we directly followed. The detailed preprocessing pipeline is shown in Additional file 2: Table S1.

### Validation method

To comprehensively evaluate the performance of the CDSKNN clustering framework, we compared it with the mainstream clustering frameworks in large-scale single-cell transcriptomics, including PARC [21], phonograph [18], and FlowGrid [22]. These frameworks, which utilize density and community discovery approaches, were assessed using their default settings. Our comparison focused on the following aspects: clustering accuracy, the accurate estimation of the number of clusters, clustering speed, and the repeatability of marker genes in different clustering results.

Specifically, we conducted a robustness test of the CDSKNN parameters using the first group datasets and validated the method's adaptability to imbalanced data ratios. Then, we assessed the method's clustering performance, computational efficiency, and its capacity to identify marker genes within the second group datasets. These datasets comprised single-cell data from various origins, featuring a range of cell quantities.

All the experiments were performed on our CentOS system with 48 CPU cores at 2.2 GHz, 250 GB of memory. For comparison under the same conditions, we conducted comparison experiments on a single CPU.

### Evaluation measurement

#### Clustering accuracy

We adopt Adjusted Rand Index (ARI) to evaluated clustering accuracy, which was based on the Rand index (RI). We represent the known cell types as R and the identified clusters as $E\left[RI\right]$ and ARI are defined as follows:

6 $$RI\left(R,E\right)=\frac{TP+TN}{TP+FP+FN+TN}$$

7 $$ARI(R,E)=\frac{RI-E\left[RI\right]}{max\left(RI\right)-E\left[RI\right]}$$

where TP is the number of true positives, TN is the number of true negatives, FP is the number of false positives, and FN is the number of false negatives. As shown in Eq. (7), where E[RI] represents the expected value. A higher ARI value indicates that the clustering result is more consistent with the actual situation.

#### Accuracy of the estimated number of cell clusters

Both CDSKNN and the three comparative methods offer streamlined approaches that do not require predefining the number of clusters, relying instead on each method's comprehensive data analysis and interpretation to determine cluster counts. To assess the accuracy of cell type quantity estimations by these methods, we calculated $deviation$ between the cell type numbers obtained from the clustering results and the benchmark cell type numbers. A positive deviation indicates an overestimation, whereas a negative one suggests an underestimation.

#### The reproducibility of marker genes

The distinct marker genes, representing the biological origins of cell clusters, are essential for comprehending the biological context of a cell type. We conducted differential gene expression analysis on the clustering results and compared them with marker genes from original studies using Jaccard similarity [41]. This comparison allowed us to evaluate the accuracy of different methods in replicating essential cell type marker genes.

## Results

### CDSKNN parameter settings

We conducted multiple experiments on the first group of datasets to test the robustness of the key parameters in the CDSKNN and selected the optimal parameter configuration. Specifically, for each parameter, we varied its settings within a certain range while keeping other parameters at their default values. To assess whether significant changes occurred, the clustering performance of CDSKNN with different parameter configurations was evaluated using the Wilcoxon signed rank test. The assessment was based on $ARI$ and $deviation$, along with considerations of time consumption. The default settings and search ranges for the main parameters are as follows:

1. $g$: the number of region partitions. The default setting is 500. The tests were conducted within the range of 200–2000 at intervals of 300.
2. ${k}_{max}$: the maximum value for the $K$ value search range. The default setting is 50. The test is conducted within $\{20, 30, 40, 50, 80, 110, 140, 170, 200\}$.
3. $res$: the resolution of the louvain algorithm during resampling. The default setting is 1.0. The tests were conducted within the range of 0.2–3.0 at intervals of 0.4.
4. $L$: the number of iterations for resampling. The default setting is 50. The tests were performed within the range of 20–200 at intervals of 30.

In relation to the number of regional partitions ($g$), ${ARI}_{med}$ indicates that the clustering outcomes demonstrate the highest alignment with the gold standard when adhering to the default configuration (${ARI}_{med}=0.66$) (Additional file 1: Fig S1a). For $g=200$, there is a 55.5% reduction in ${time}_{med}$ (${time}_{med}=1.318$) compared to $g=500$ (${time}_{med}=2.964$), but the ${ARI}_{med}$ dropped significantly (${ARI}_{med}^{g=200}=0.5$). Considering all these factors, we believe that accurately identifying cell types takes precedence over minimizing computational costs. Consequently, we have established the optimal value for $g$ to be 500.

Concerning the exploration of the K value in the KNN graph structure, we established the lower limit at 3 and broadened the exploration range by adjusting the upper bound, denoted as ${k}_{max}$ practical terms. There is no requirement for an excessively extensive exploration range, as a higher $K$ value implies a smoother graph structure, facilitating easier point connectivity and potentially overlooking localized structures within the dataset. When ${k}_{max}$ is 50 or greater, the median values of $ARI$ and $deviation$ remain constant ($AR{I}_{med}=0.66$, ${deviation}_{med}=-7$) (Additional file 1: Fig S1b). For $K$ values less than 50, we observed an enhancement in the deviation results compared to the scenario with $K$ at 50 ($deviatio{n}_{med}\in \left[-5, -4\right]$). Consequently, we opt for a search window width of $K$, ranging from 3 to 50.

Regarding the configuration of the resolution ($res$) in the context of the louvain algorithm during the resampling process, both the deviation and time consumption exhibit insensitivity to changes in the parameter, demonstrating no significant fluctuations (${deviation}_{med}\in \left[-7, -5\right], {time}_{med}\in \left[2.964, 3.329\right] mins$) (Additional file 1: Fig S1c). Therefore, we set the optimal value for $res$ to 1.

Concerning the number of resampling iterations ($L$), the most prominent observation is the increase in time consumption as the number of sampling iterations increases $(L\in \left[20, 200\right] , {time}_{med}\in \left[1.458, 15.58\right] mins$) (Additional file 1: Fig S1d). Additionally, there is no noteworthy fluctuation in clustering accuracy $(ARI_{med} \in \left[ {0.47,{ }0.66} \right],deviation_{med} \in \left[ { - 7,{ } - 6} \right]$), with the peak occurring at$L=50$. Consequently, we choose the optimal value for $L$ to be 50.

In the following work, we will proceed with performance comparisons against other methods based on the selected optimal parameter combination.

### CDSKNN outperforms other methods in clustering data with highly imbalanced cell ratios

In scRNA-seq data, imbalances often lead to an overemphasis on certain cell types while neglecting others. The imbalance ratio (IR) serves as a metric for measuring data imbalance and represents the ratio of the number of cells in the largest cluster to the number in the smallest cluster [42]. A higher IR indicates greater data imbalance. We applied CDSKNN and three other methods to the first group datasets and evaluated the robust clustering capabilities of these methods by displaying the IR values for the first dataset ($IR\in [48, 34967]$). To conduct a comprehensive performance comparison among the methods, we performed experiments on each dataset using multiple clustering results, with the PC numbers ranging from 5 to 30 at intervals of 5.

**Fig. 2:**
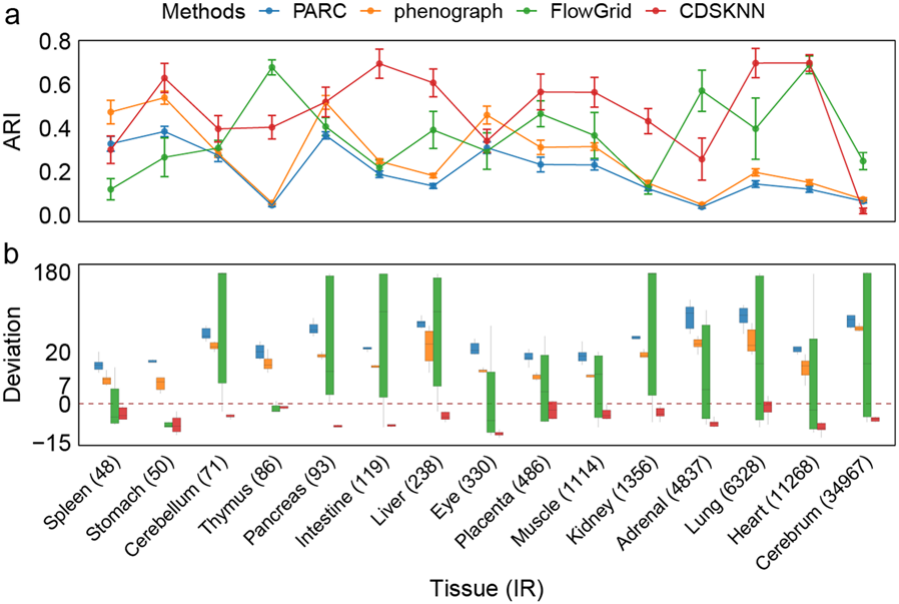
Performance comparison of CDSKNN against 3 competitive tools with increasing IR datasets ranging from 48 to 34,967. **a** Evaluation with $ARI$. The actual values are presented as the mean $\pm$ standard deviation. **b** Evaluation with $deviation$. The horizontal lines in the box represent median values, with whiskers extending to the farthest data point within a maximum of 1.5 × interquartile range

Among the 15 datasets characterized by varying IRs, CDSKNN surpasses the other three methods in 10 tissues, yielding an $AR{I}_{med}$ of 0.46, in contrast to 0.19 (PARC), 0.261 (phenograph), and 0.302 (FlowGrid) for the respective methods (Fig. 2a). Specifically, CDSKNN outperforms PARC in 13 tissues, excluding the cerebrum and spleen, with a clustering consistency difference ranging from 0.032 to 0.58. In datasets with high IRs, such as the cerebrum and adrenal, CDSKNN shows marginally less clustering consistency than FlowGrid but outperforms it in low-IR tissues like the spleen, stomach, and cerebellum. Additionally, CDSKNN surpasses phenograph in 11 tissues, aside from the spleen, eye, pancreas, and cerebrum, with the difference in clustering consistency varying from 0.08 to 0.566. Furthermore, we performed a comparison to determine if the cell type quantities identified by various methods matched the benchmark cell type numbers in the study, aiming for biologically interpretable results. The findings revealed that CDSKNN tended to underestimate the number of cell types, with $deviatio{n}_{med}$ ranging from $\left[-11.5, -0.5\right]$ and $deviatio{n}_{sd}$ ranging from [0.894,3.94]. Conversely, PARC and phenograph tended to overestimate the number of cell types in tissue data with diverse IRs (Fig. 2b). Compared to the established gold standard, these two methods estimate cell type numbers to be greater within the ranges of $\left[15, 35\right]$ and $\left[8.5, 29\right]$. FlowGrid’s $deviatio{n}_{med}$ spans a wide range across all tissues, varying from $\left[-8.5, 60.5\right]$, with $deviatio{n}_{sd}$ ranging from $\left[1.83, 82.3\right]$, indicating a notably unstable performance.

Overall, these findings demonstrate that CDSKNN is effective at addressing the imbalance within single-cell data. By confronting data characterized by imbalanced intercluster proportions, CDSKNN can reasonably perform clustering to a certain extent and provide a more accurate estimation of the number of cell types compared to other methods.

### CDSKNN exhibits preferable scalability and good performance on large-scale datasets

We applied CDSKNN alongside three other clustering frameworks to diverse datasets sourced from various origins and tissues, including lung tissue, neurons, and peripheral blood, among others (Table 1, Additional file 2: Table S1). These datasets cover a broad spectrum of cell counts, ranging from 12,954 to 1,462,702 cells, and include three datasets comprising millions of single cells.

**Fig. 3:**
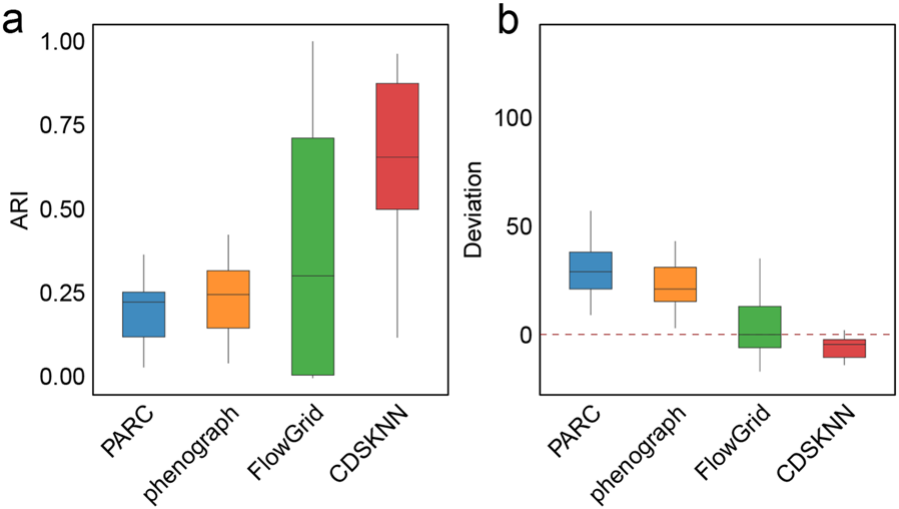
Overall clustering performance comparison of CDSKNN and existing methods on diverse datasets sourced from various origins and tissues. Evaluation with $ARI$ (**a**) and $deviation$ (**b**). The horizontal lines in the box represent median values, with whiskers extending to the farthest data point within a maximum of 1.5 × interquartile range

**Fig. 4:**
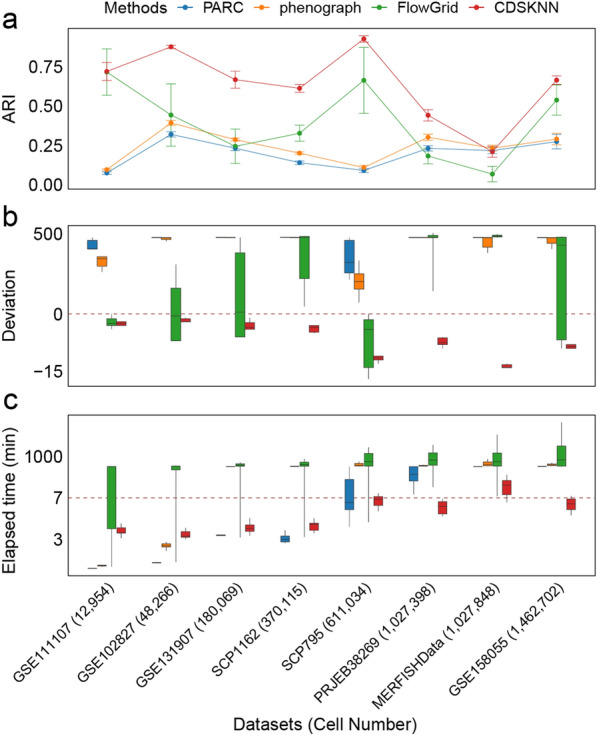
Comparison of the CDSKNN and existing methods for datasets with different numbers of cells. **a** Evaluation with $ARI$**.** The actual values are presented as the mean $\pm$ standard deviation; **b**-**c** Evaluation with $deviation$ (**b**) and elapsed time (minutes) (**c**). The horizontal lines in the box represent median values, with whiskers extending to the farthest data point within a maximum of 1.5 × interquartile range. For the sake of clarity, we truncated and compressed the y-axis

When the number of principal components (PCs) is set to 5, the distribution of clustering results from different methods within the embedding space further validates the effective clustering by CDSKNN (Additional file 1: Fig S2), with its predicted number of clusters closely matching the benchmark cell types number. In contrast, the clustering outcomes of the other three methods often demonstrate an effect of over-clustering [43]. Integrating results across all principal component numbers, CDSKNN consistently outperforms the other methods in clustering consistency and accurately estimating cell type quantities (Fig. 3). For instance, in the GSE102827 (48,266 cells), CDSKNN achieved an $AR{I}_{med}$ of 0.882, signifying an enhancement ranging from 0.332 (PARC) to 0.427 (FlowGrid) relative to other methodologies (Fig. 4a). The margin of $deviation$ between the estimated quantities of cell types and the reference standard remains within the interval [-2, -1], demonstrating stability that surpasses other methods (Fig. 4b). Similar trends are evident in the GSE131907 (180,069 cells) and SCP1162 (370,115 cells) datasets. Furthermore, the CDSKNN has demonstrated significant effectiveness in managing large-scale single-cell datasets. For SCP795 (611,034 cells), $AR{I}_{med}$ of CDSKNN reached 0.949, indicating an improvement over phenograph ($AR{I}_{med}=0.116$) and PARC ($AR{I}_{med}=0.099$). In 3 datasets comprising millions of single cells, the comparative methods exhibited deviation ranges in estimating cell type quantities of $[22, 37]$ (PARC), $[20, 31.5]$ (phenograph), and $[18, 207]$ (FlowGrid) (Fig. 4b). In contrast, the CDSKNN exhibits enhanced stability with a narrower margin of error ($deviation\in [-14,-7.5]$), emphasizing its applicability and dependability in the analysis of large-scale single-cell data.

Additionally, we meticulously documented the runtime of each algorithm. The time consumption of CDSKNN did not significantly increase with the number of cells, but remained stable (Fig. 4c). While handling 3 datasets comprising millions of single cells, the median level of time consumption for CDSKNN was only 6.18–8.22 min, representing a 33.3% to 99% reduction in runtime compared to the other three methods. FlowGrid's average runtime reached 1,403 min when processing 1.46 million data points, whereas CDSKNN required only 6.33 min.

### CDSKNN has better clustering stability with different numbers of principal components

**Fig. 5:**
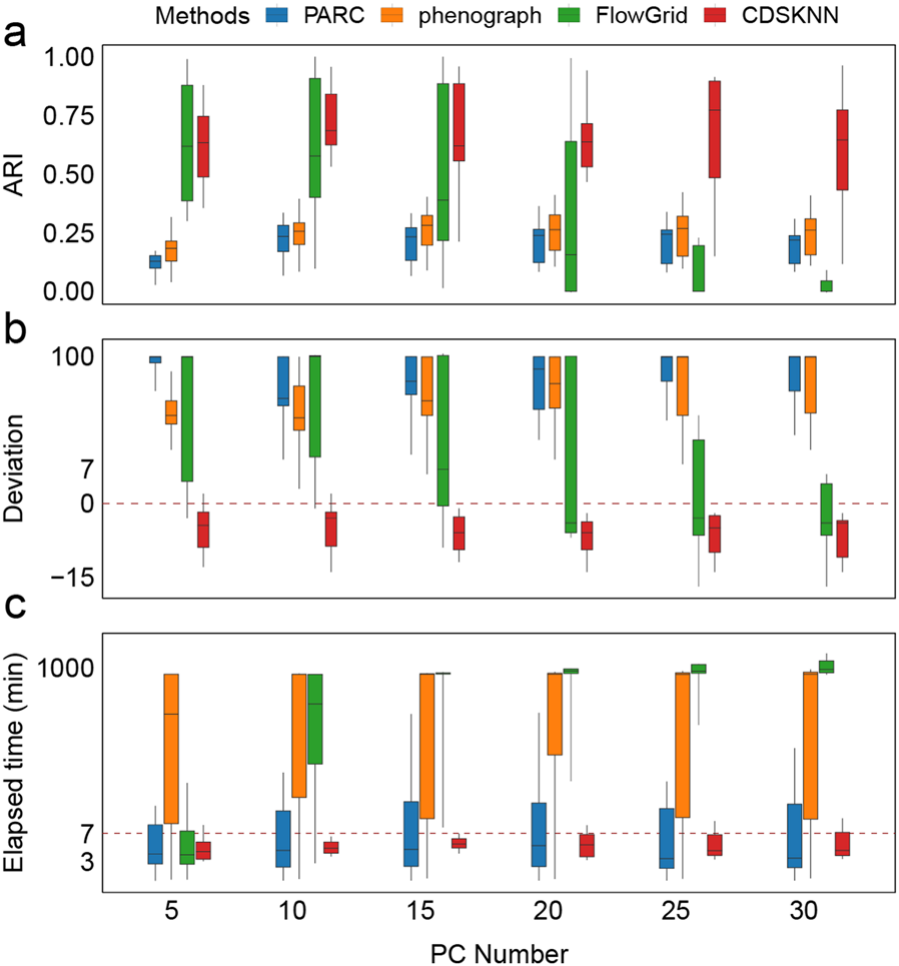
Comparison of the CDSKNN and existing methods under different numbers of principal components. **a**-**c** Evaluation with $ARI$ (**a**), $deviation$ (**b**) and elapsed time (minutes) (**c**). The horizontal lines in the box represent median values, with whiskers extending to the farthest data point within a maximum of 1.5 × interquartile range. For the sake of clarity, we truncated and compressed the y-axis

Based on the results obtained from datasets of varying sizes, we conducted a comparative analysis of clustering performance across different numbers of principal components (PCs). Overall, the CDSKNN exhibited superior stability to that of the other three methods (Fig. 5). Both the efficiency of clustering and the runtime remain relatively steady despite changes in the number of PCs. As the number of PCs increases, the CDSKNN algorithm displays slight fluctuations ($AR{I}_{med}\in \left[0.62, 0.772\right]$, ${deviation}_{med}\in \left[-6,-3\right]$), while the time consumption remains stable at approximately 3–7 min. Despite its shorter runtime, PARC demonstrates inadequate clustering performance, whereas phenograph also lack competitiveness.

Of particular note is FlowGrid, which shows comparable performance to CDSKNN when the number of PCs is 5 (${ARI}_{med}^{(FlowGrid)}=0.618$, ${ARI}_{med}^{(CDSKNN)}=0.633$) and consumes less time (Fig. 5a). However, as the number of PCs increases to 20, FlowGrid experiences a sharp decline in clustering performance ($AR{I}_{med}=0.15$), accompanied by a significant increase in time consumption (${time}_{med}=527.7 mins$) (Fig. 5c). In contrast, CDSKNN consistently exhibits strong clustering performance ($AR{I}_{med}=0.636$) and relatively minimal computational time (${time}_{med}=5.385 mins$). These findings suggest that CDSKNN is more adept than FlowGrid at capturing the intricate data structures present within single-cell datasets. Similarly, the comparison results for $deviation$ underscore the ability of CDSKNN to provide stable cell type estimation (Fig. 5b).

### CDSKNN can reproduce marker genes of major cell types

The distinct marker genes, representing the biological origins of cell clusters, are pivotal for grasping the biological essence of a cell type. Through differential gene expression (DGE) analysis grounded on clustering outcomes, and benchmarking against marker genes validated in original research (log2 Fold change > 2, Adjust P-value < 0.01), we confirmed the clustering technique's proficiency in mirroring marker genes of major cell types, quantitatively assessed by Jaccard similarity ($jac$).

**Fig. 6:**
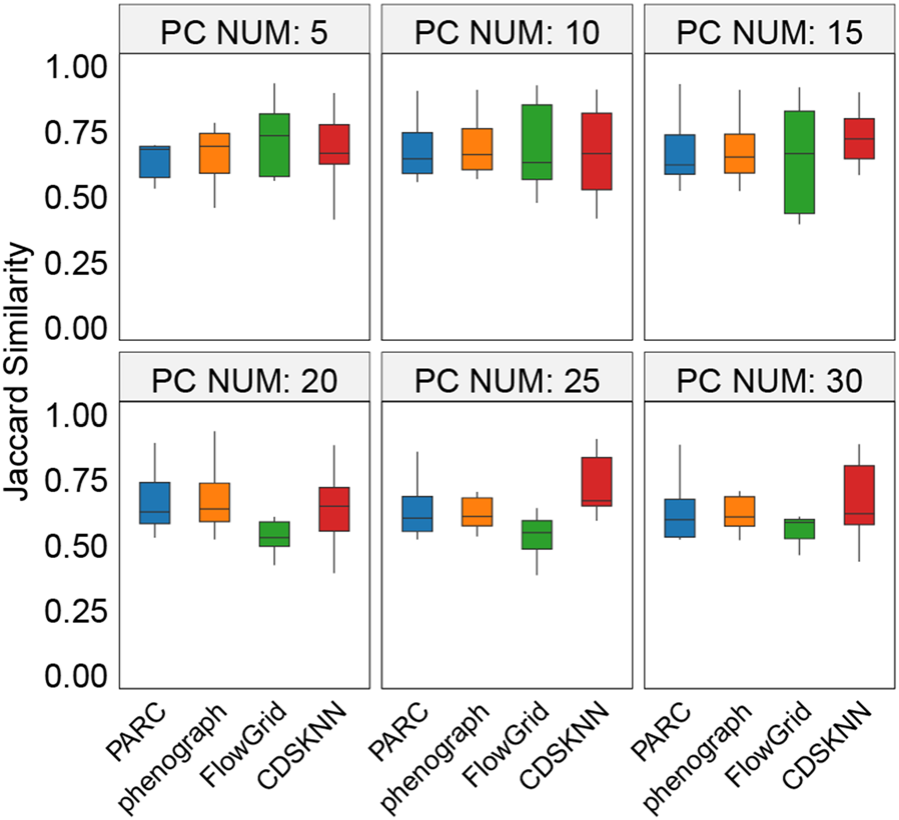
Comparison of the Jaccard similarity between clustering marker genes and benchmark cell type-specific marker genes based on different clustering methods across second group datasets

When the number of PCs is set to 5, the ${jac}_{med}$ of the DGE results obtained from CDSKNN compared to the benchmark is slightly lower than that of FlowGrid (Fig. 6). Nonetheless, CDSKNN demonstrates remarkable performance in other evaluations. As the number of PCs increases, CDSKNN's benefits become more evident, with the ${jac}_{med}$ rising from 0.668 to 0.723. Additionally, we emphasized the $jac$ between the clustering marker genes and benchmark results across three datasets, each boasting over a million data points. CDSKNN demonstrates a tendency to form fewer clusters, with each cluster corresponding to a specific major cell type (Additional file 1: Fig S3). This is confirmed by comparing the expression heatmaps of the top 2 marker genes with the highest fold changes in each benchmark cell type across various clusters (Additional file 1: Fig S4). In contrast, the other three methods show a pattern of over-clustering and over-representation, where a single cluster might include marker genes from multiple cell types.

## Discussion

With the increasing profundity of sequencing and the increasing coverage of throughput, single-cell data amplified on an unparalleled scale necessitates novel computational tools for effective management of the magnitude and intricacy of single-cell datasets. By constructing a stable KNN graph structure, we proposed CDSKNN, an unsupervised cluster integration approach suitable for cell grouping. To address large-scale single-cell data, CDSKNN is primarily grounded in two key components: i) partitioning to reduce computational complexity and eliminate outliers; ii) data-centric construction of a stable KNN graph structure. To leveraging the stability of the graph structure, we apply cluster evaluation metrics to ascertain the best fitting cluster resolution. Validation revealed that initial data partitioning expedites the analysis pipeline, while a stable graph structure notably fortifies cell clustering. Firstly, we undertook parameter testing and comparative validation using a single-cell dataset sourced from the human fetal atlas [31]. In comparison with existing methodologies, CDSKNN effectively captures data structures, executes proficient clustering, and delivers accurate estimations of cell type quantities, particularly when dealing with highly imbalanced data scenarios. Secondly, we conducted performance comparisons across multiple single-cell datasets derived from diverse studies and library construction methods. This broader assessment highlights the flexibility of CDSKNN in adapting to the inherent complexities of these datasets, as well as its ability to effectively preserve marker genes associated with major cell types, thereby providing a biologically meaningful representation of the data. Thirdly, as the number of cells rapidly increases, CDSKNN demonstrates efficient clustering within a minimal time period, highlighting its superior scalability in managing extensive datasets relative to alternative approaches.

The above results underscore the practical application of CDSKNN as a single-cell data clustering tool within complex and diverse biological systems. With the increasing throughput of single-cell sequencing, efficient data management and effective handling of imbalances are crucial for single-cell data analysis. CDSKNN, serving as a flexible framework for automated clustering, provides rapid data-driven clustering for researchers with different biological backgrounds, particularly demonstrating high computational efficiency in large-scale datasets. Additionally, it effectively addresses imbalanced data issues, offers a more reliable underlying representation of biological processes.

While the CDSKNN offers a promising strategy for clustering single-cell data, recognizing its limitations is crucial. A notable challenge is its use of partition clustering algorithm for region partitioning, which might not effectively capture the non-linear relationships in the increasingly complex single-cell sequencing data. Additionally, the method for choosing the optimal clustering resolution currently relies solely on cluster quality assessment. Incorporating differential testing between clustering results could provide a more refined strategy for identifying the best resolution. Finally, the exclusive use of ARI for evaluating clustering performance may not comprehensively reflect result accuracy. Expanding the evaluation framework to include a broader array of metrics, while also considering the interpretability of clustering outcomes and their influence on further analyses, can lead to a more thorough and precise evaluation of the method's effectiveness.

## Conclusions

We propose CDSKNN^XMBD^, an unsupervised clustering framework designed to group cells by constructing a stable KNN graph structure. Compared to existing methods, CDSKNN accurately captures the data structure in highly imbalanced scenarios, achieving efficient clustering and accurately estimating the number of cell types. CDSKNN is applicable to single-cell data from diverse biological backgrounds and can efficiently and accurately cluster millions of single cells in a short time, highlighting its universality and scalability. Moreover, CDSKNN showed consistent clustering performance across various numbers of features, offering a more dependable underlying representation of biological processes.

### Supplementary Information

**Additional file 1: Fig S1**. Robustness testing was conducted on the key parameters within CDSKNN. **Fig S2.** UMAP visualization of second group datasets, colored according to benchmark cell type labels and clustering results from 4 frameworks. **Fig S3.** The Jaccard similarity between clustering marker genes and benchmark cell type-specific marker genes across three datasets, each with millions of data points. **Fig S4.** In three million-cell datasets, the heatmap shows the expression of marker genes for benchmark cell types across the results of different clustering frameworks.

**Additional file 2: Table S1**. General information and detailed preprocessing pipeline for the validation single-cell sequencing datasets.

## Data Availability

The R implementation of CDSKNN is available for academic use on GitHub (https://github.com/renjun0324/CDSKNN). All datasets utilized in this study are sourced from previously published works and are publicly accessible. Detailed information about these datasets is provided in Table 1 and Additional file 2: Table S1, while Table S1 contains the complete preprocessing pipeline.
