# Supplementary material for: CDSKNNXMBD: a novel clustering framework for large-scale single-cell data based on a stable graph structure
Source: J Transl Med. 2024 Mar 3;22:233. doi: 10.1186/s12967-024-05009-w (PMC10910752; doi:10.1186/s12967-024-05009-w)
Supplement: Supplementary file 1 — Additional file 1: Fig S1. Robustness testing was conducted on the key parameters within CDSKNN. Fig S2. UMAP visualization of second group datasets, colored according to benchmark cell type labels and clustering results from 4 frameworks. Fig S3. The Jaccard similarity between clustering marker genes and benchmark cell type-specific marker genes across three datasets, each with millions of data points. Fig S4. In three million-cell datasets, the heatmap shows the expression of marker genes for benchmark cell types across the results of different clustering frameworks. [file 12967_2024_5009_MOESM1_ESM.docx]

# Supplementary Figure


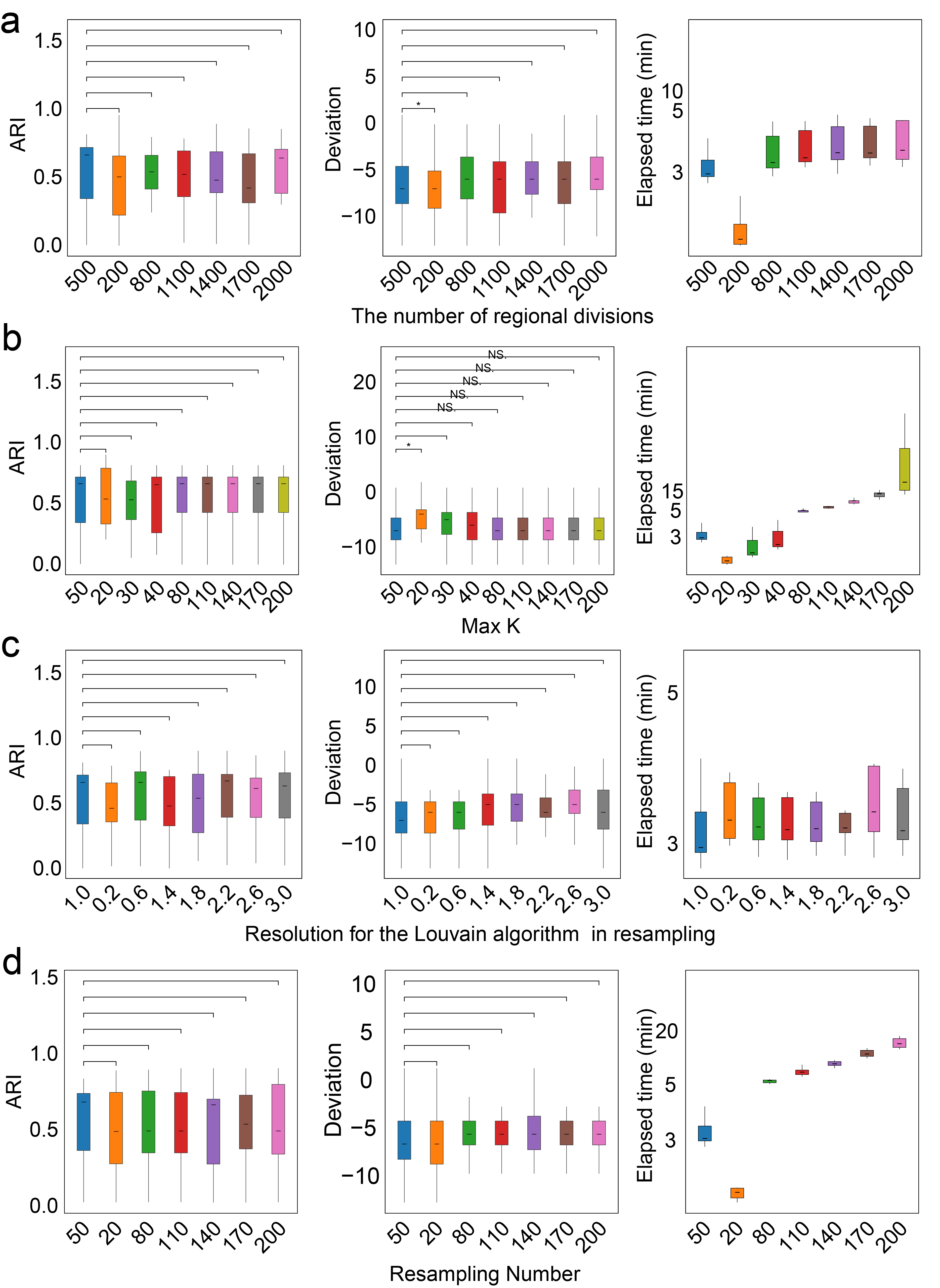


**Fig S1.** **Robustness testing was conducted on the key parameters within CDSKNN.** For each specific parameter, a designated range was tested while maintaining the other parameters at default values. The Wilcoxon signed rank test was used to determine significant changes in the predictive performance of CDSKNN across different parameter settings. The parameters subjected to robustness testing encompass the following aspects: (a). Quantity of partitions in the region (K); (b). Maximum value of the search window for the K value; (c). Resolution of the Louvain algorithm during resampling (res); (d). Number of iterations during resampling (L). The evaluation metrics of each parameter include $ARI$, $deviation$, and elapsed time (minutes).


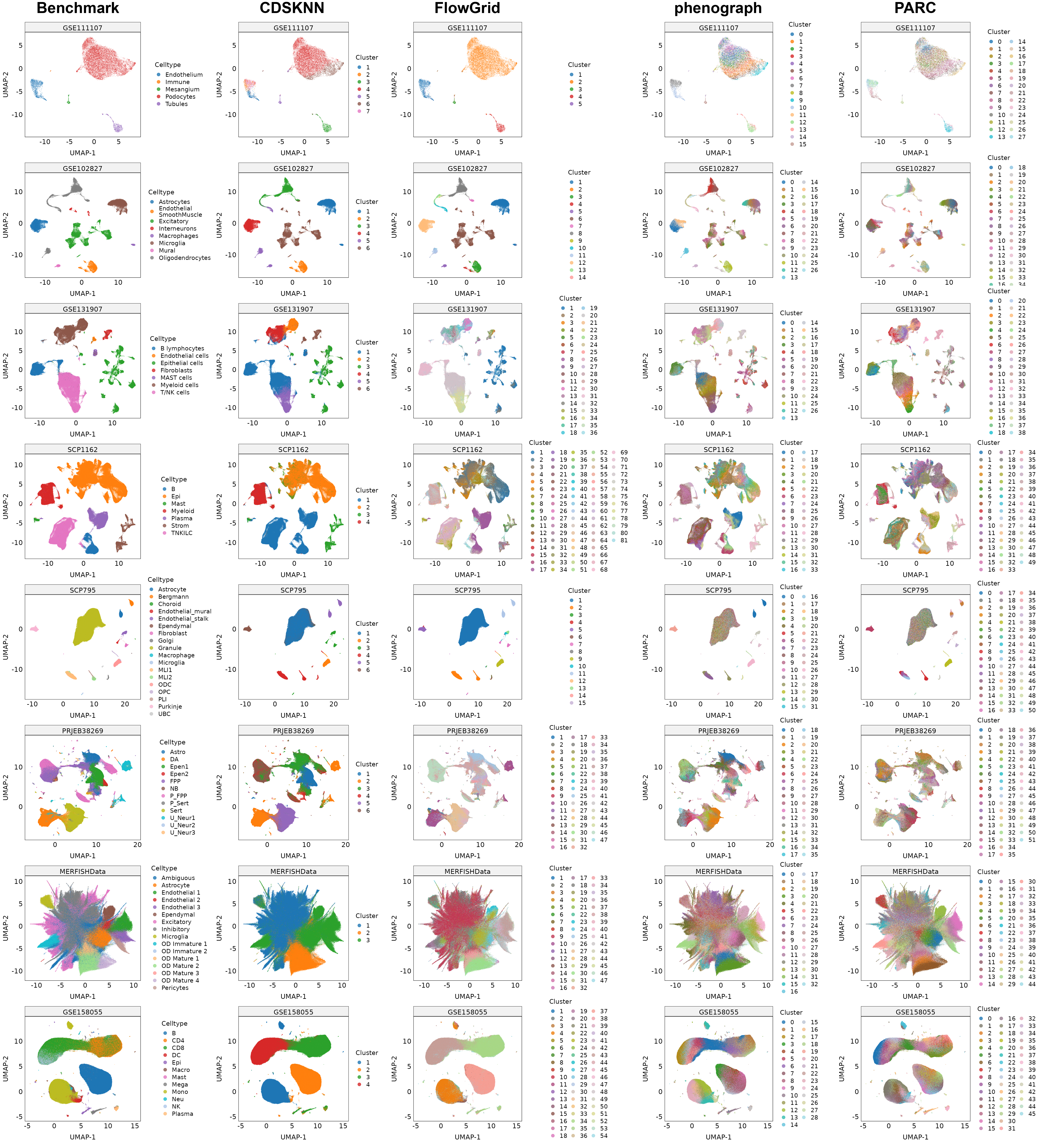


**Fig S2. UMAP visualization of the second group dataset, color-coded by benchmark cell type labels and clustering outcomes from four frameworks.**


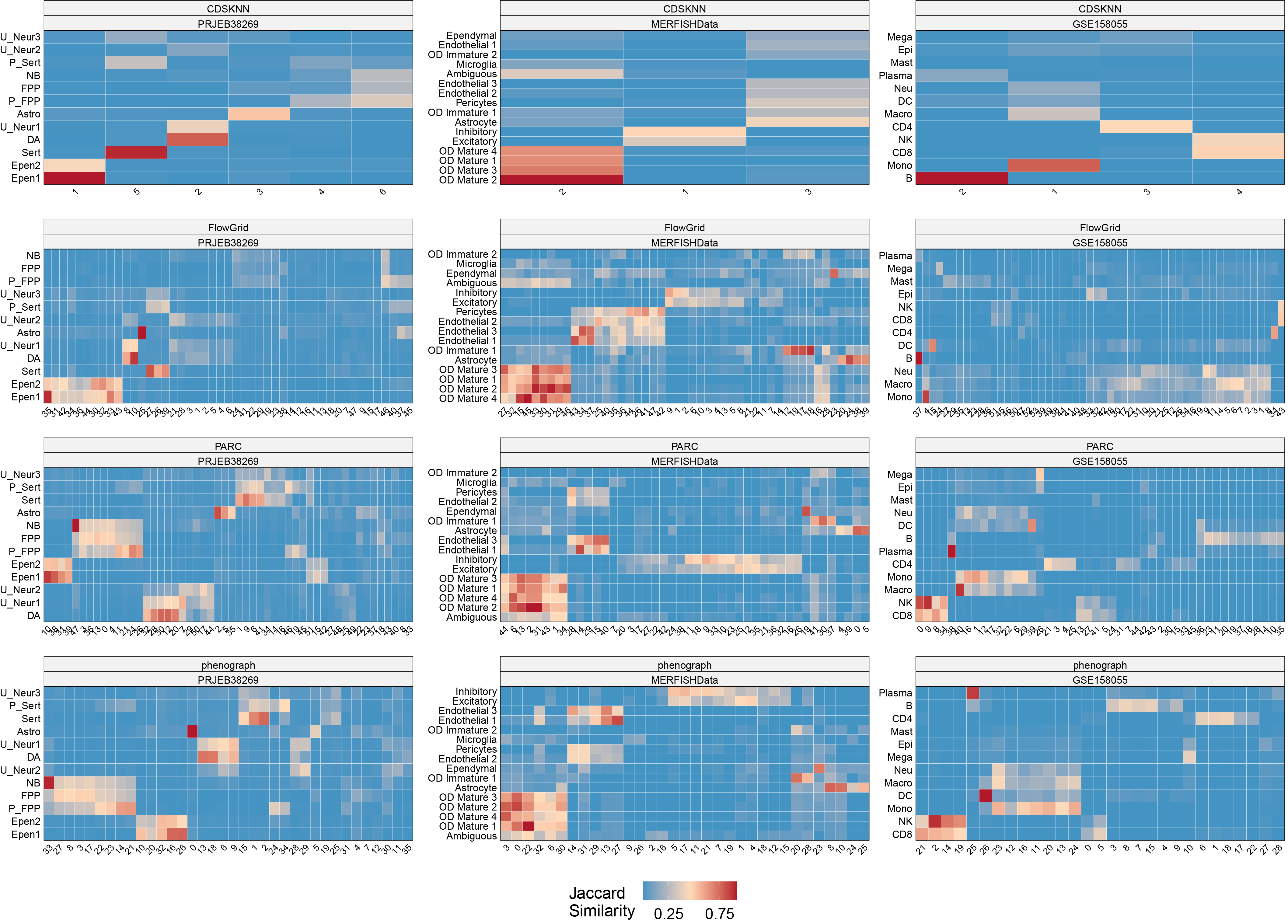


**Fig S3. The Jaccard similarity between clustering marker genes and benchmark cell type-specific marker genes across three datasets, each with millions of data points.** Rows indicate benchmark cell types, and columns show the results of different clustering methods.


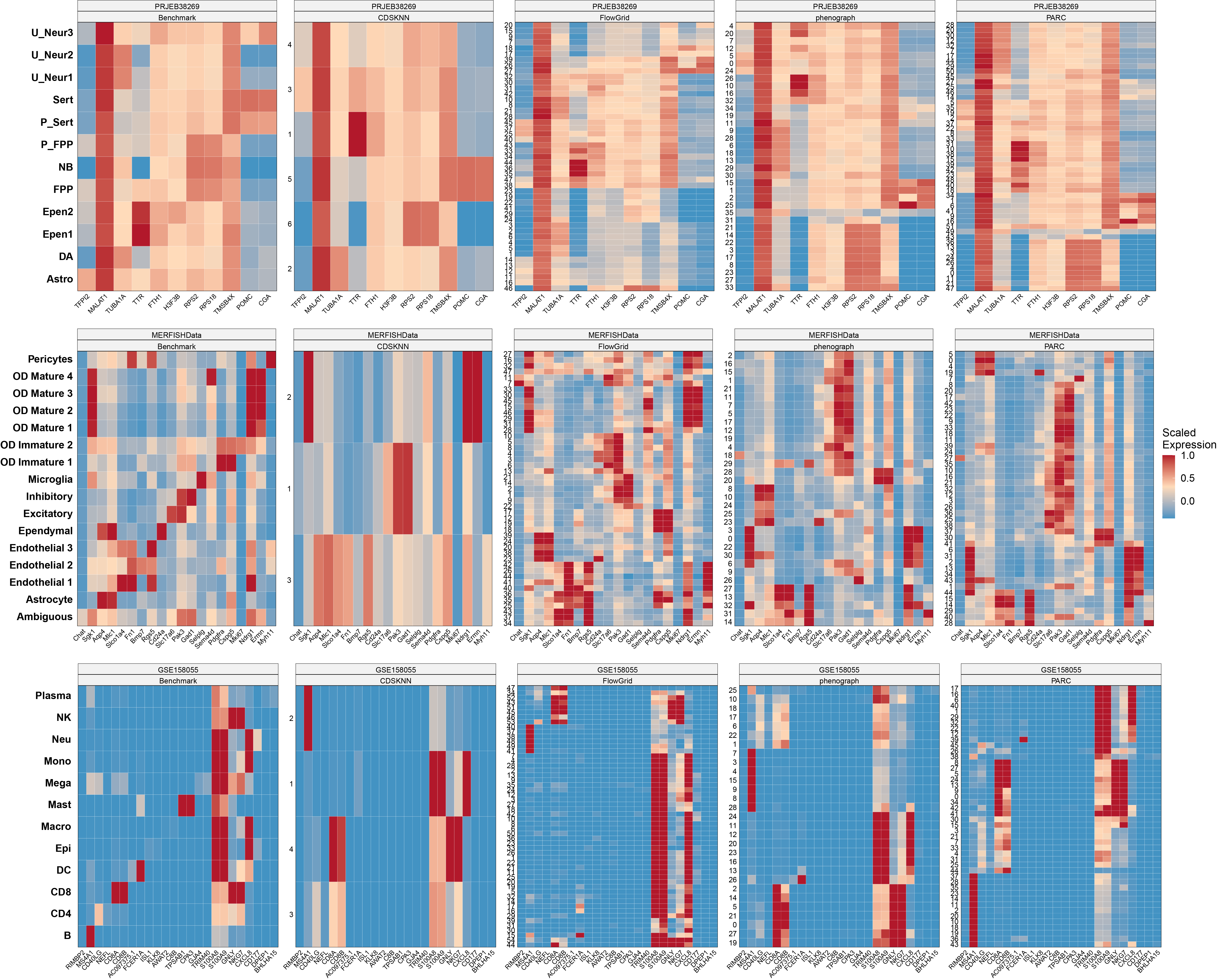


**Fig S4. In three million-cell scRNA-seq datasets, the heatmap shows the expression of marker genes for benchmark cell types across the results of different clustering methods.**
